# Supplementary material for: MetaRibo-Seq measures translation in microbiomes
Source: Nat Commun. 2020 Jun 29;11:3268. doi: 10.1038/s41467-020-17081-z (PMC7324362; doi:10.1038/s41467-020-17081-z)
Supplement: Supplementary file 10 — Supplementary Data 7 [file 41467_2020_17081_MOESM10_ESM.zip › File2/Confidence_High_Taxonomy/443400_out.krona.html]

Javascript must be enabled to view this page.

members
magnitude
magnitudeUnassigned
count
unassigned
taxon
rank

443400\_out

92

superkingdom
92
2

phylum
92
1239

class
186801
92

8
186802
92
order

SRS012273\_contig\_number\_contig-100\_965.278045SRS015663\_contig\_number\_12533SRS015854\_contig\_number\_19389SRS065504\_contig\_number\_30308SRS104485\_contig\_number\_5684SRS1055049\_contig\_number\_contig-100\_1077.98245SRS893270\_contig\_number\_21993SRS893366\_contig\_number\_contig-100\_2251.139524

family
40
31979

1
1649459
genus


SRS100021\_contig\_number\_11977
species
154046
1

1485
38
genus

38
59620
species

SRS011134\_contig\_number\_46118SRS011239\_contig\_number\_24286SRS013800\_contig\_number\_contig-100\_1917.142216SRS014235\_contig\_number\_contig-100\_1084.267509SRS014287\_contig\_number\_13154SRS014979\_contig\_number\_29413SRS015264\_contig\_number\_contig-100\_1051.123776SRS015579\_contig\_number\_25805SRS017521\_contig\_number\_contig-100\_216.272359SRS018427\_contig\_number\_28195SRS018656\_contig\_number\_contig-100\_605.79697SRS018817\_contig\_number\_13757SRS019161\_contig\_number\_contig-100\_1245.238511SRS022137\_contig\_number\_22216SRS022609\_contig\_number\_contig-100\_790.274270SRS023715\_contig\_number\_contig-100\_790.176565SRS024435\_contig\_number\_contig-100\_1168.253203SRS024492\_contig\_number\_18326SRS042284\_contig\_number\_7082SRS043001\_contig\_number\_9305SRS043667\_contig\_number\_contig-100\_1091.46656SRS048060\_contig\_number\_contig-100\_865.84427SRS048164\_contig\_number\_33431SRS048870\_contig\_number\_contig-100\_1511.129388SRS049995\_contig\_number\_contig-100\_559.222025SRS058070\_contig\_number\_contig-100\_1041.189594SRS062427\_contig\_number\_contig-100\_820.78219SRS063518\_contig\_number\_28694SRS075773\_contig\_number\_contig-100\_827.239546SRS1055022\_contig\_number\_contig-100\_376.149701SRS142712\_contig\_number\_contig-100\_753.47894SRS143598\_contig\_number\_19472SRS144714\_contig\_number\_contig-100\_852.173234SRS147139\_contig\_number\_contig-100\_1344.213986SRS148319\_contig\_number\_contig-100\_311.66372SRS148970\_contig\_number\_1280SRS149879\_contig\_number\_27585SRS893230\_contig\_number\_contig-100\_644.81447

1
580596
genus


SRS013951\_contig\_number\_29842
species
2292199
1

family
12
541000

3
12
1263

SRS064645\_contig\_number\_230SRS077454\_contig\_number\_19798SRS144183\_contig\_number\_contig-100\_1125.220086
genus


SRS1054928\_contig\_number\_679
species
165186
1


SRS147271\_contig\_number\_contig-100\_483.244602
species
2292251
1

2293187
1

SRS050422\_contig\_number\_contig-100\_61.201965
species

1
2293234

SRS1041033\_contig\_number\_29104
species

3
657323

SRS042628\_contig\_number\_28520SRS042966\_contig\_number\_20494SRS075341\_contig\_number\_25012
species

2293236
1

SRS893259\_contig\_number\_contig-100\_705.54536
species

2293158
1
species

SRS055982\_contig\_number\_19699

2
186803
32
family

SRS020328\_contig\_number\_contig-100\_963.211551SRS049896\_contig\_number\_21081

397288
1
species

SRS143417\_contig\_number\_22721

genus
1
207244

species

SRS144297\_contig\_number\_contig-100\_460.107331
649756
1


SRS063127\_contig\_number\_contig-100\_919.40449SRS104311\_contig\_number\_contig-100\_1813.256961SRS144537\_contig\_number\_36244
species
1898203
3

genus
2
2005359


SRS1041031\_contig\_number\_30866SRS1041140\_contig\_number\_14584
species
39482
2

1407607
1
genus

1806509
1

SRS893170\_contig\_number\_8153
species

genus
189330
2


SRS016018\_contig\_number\_11234
species
1
88431

1
2293098
species

SRS049900\_contig\_number\_contig-100\_895.121913

39491
1
species

SRS054590\_contig\_number\_1784

genus
1
841

species

SRS098881\_contig\_number\_20225
1897017
1

18
572511
2

SRS014313\_contig\_number\_contig-100\_359.52940SRS015890\_contig\_number\_24211
genus

species

SRS148159\_contig\_number\_2811
2292287
1

1
33038

SRS140492\_contig\_number\_27190
species

765821
10
species

SRS014855\_contig\_number\_12118SRS015794\_contig\_number\_contig-100\_738.90572SRS018984\_contig\_number\_27863SRS045713\_contig\_number\_10920SRS053356\_contig\_number\_47884SRS063370\_contig\_number\_contig-100\_48.87913SRS078176\_contig\_number\_4320SRS1041164\_contig\_number\_3462SRS1055083\_contig\_number\_2962SRS971275\_contig\_number\_43390

40520
1

SRS044535\_contig\_number\_contig-100\_501.147432
species

1805476
3

SRS014459\_contig\_number\_contig-100\_739.236781SRS015782\_contig\_number\_contig-100\_924.260986SRS097958\_contig\_number\_25298
species
